# Physiological and molecular responses of two contrasting drought resistance pistachio interspecific hybrid rootstocks

**DOI:** 10.3389/fpls.2025.1515819

**Published:** 2025-04-22

**Authors:** Mozhdeh Osku, Silvia Procino, Isabella Mascio, Monica Marilena Miazzi, Gaetano Alessandro Vivaldi, Danilo Vona, Valentina Fanelli, Mahmoud Reza Roozban, Saadat Sarikhani, Mohammad Mehdi Arab, Mohammad Akbari, Kourosh Vahdati, Cinzia Montemurro

**Affiliations:** ^1^ Department of Horticulture, Faculty of Agricultural Technology (Aburaihan), University of Tehran, Tehran, Iran; ^2^ Department of Soil, Plant and Food Sciences (DiSSPA), University of Bari Aldo Moro, Bari, Italy; ^3^ School of Biotechnology, College of Science, University of Tehran, Tehran, Iran; ^4^ Royeshe Sabze Farda Research Center (Pistat), Nazari Business Group, Tehran, Iran; ^5^ SINAGRI S.r.l. Spin Off of University of Bari Aldo Moro, Bari, Italy

**Keywords:** clonal hybrids, gene expression, osmolyte accumulation, root architecture, water withholding

## Abstract

Pistachio (*Pistacia vera* L.) is a valuable nut crop that faces significant challenges due to drought stress, which can severely impact its growth, yield, and quality. Understanding the physiological and molecular mechanisms underlying drought tolerance is crucial for developing resilient pistachio rootstock. In this study, among nine-month-old saplings of seven clonal interspecies hybrids of *Pistacia atlantica* × *Pistacia integerrima*, two contrasting hybrids, ‘C4-2’ (sensitive) and ‘C9-4’ (resistant), were assessed for their morphological, physiological and molecular responses to 30 days of withholding irrigation. Water withholding induced alterations in root architecture in the resistant clone, accompanied by an increase in compatible solutes, including glycine betaine, proline, and total soluble carbohydrates. Enzyme activities of guaiacol peroxidase (GPX) and catalase (CAT) were elevated in the resistant clone under water stress. Both clones exhibited increased levels of malondialdehyde (MDA) and hydrogen peroxide (H_2_O_2_) during the stress period, with these changes being more pronounced in C4-2 compared to C9-4. In the resistant clone, both *CDPK* and *ZEP* genes were upregulated, suggesting their role in enhancing stress signaling and osmotic regulation under drought stress. The upregulation of *CDPK* indicates its involvement in calcium-mediated signaling pathways, which likely contribute to improved drought tolerance. Similarly, *DHN* expression was strongly influenced by *CDPK* activity, further emphasizing its role in maintaining cellular integrity during stress conditions. The findings provide valuable insights for developing more resilient pistachio rootstocks capable of thriving in water-limited environments. Specifically, C9-4 demonstrated significant drought tolerance in this study. Nonetheless, further research is necessary to validate the broader applicability of these findings and to evaluate its performance across various stress environments.

## 1 Introduction

Pistachio (*Pistacia vera* L.), a member of the Anacardiaceae family, is mainly cultivated in semi-arid and arid regions and it encompasses at least 11 distinct species (Pakzad et al., 2019). Iran has been a prominent contributor to the global pistachio industry for many years, and its agricultural heritage in this field dates back approximately 3000-4000 years (Esmaeilpour et al., 2016). Pistachios are valued for their nutrition, health benefits, and economic importance. Rich in unsaturated fats, proteins, fiber, vitamins, minerals, and antioxidants, they are commonly eaten raw, salted, or roasted (Yıldız et al., 1998).

The pistachio is a semi drought-resistant nut tree that can be cultivated in both rainfed and irrigated conditions. However, due to its significant water requirement, deficit irrigation is commonly practiced in most commercial orchards. Despite its resistance to drought and salinity, drought stress remains the most critical factor limiting pistachio growth and productivity in Iran, significantly affecting their performance (del Carmen Gijón et al., 2011; Raoufi et al., 2021). Since understanding the mechanisms of drought tolerance and developing new pistachio rootstocks with enhanced resistance to drought stress have become key objectives for plant biologists and crop breeders, the study investigates the morphological, physiological and molecular mechanisms behind drought resistance in pistachio hybrids, focusing on identifying resilient rootstocks for water-limited environments.

Rootstocks play a crucial role in conferring resistance and tolerance to stresses in horticultural and fruit trees (Opazo et al., 2020). In particular, root morphology and distribution in dry regions significantly affect drought tolerance as roots anchor plants to the soil and help them respond to environmental stress (Yang et al., 2020). Plants adapt the architecture of their root system to cope with stress by adjusting root length, spread, and number (Yıldırım et al., 2018). However, despite its importance, the root system has received limited attention due to its inaccessibility (Eshel and Beeckman 2013). Under water-deficit conditions, roots may increase growth or alter their architecture to enhance water uptake (Fang and Xiong, 2015), while the shoot system regulates water loss, slow growth, and produces solutes to maintain cell pressure. At the same time, drought stress triggers the accumulation of reactive oxygen species (ROS), which can damage cells, though antioxidant enzymes help mitigate ROS damage, contributing to drought resistance (Laxa et al., 2019).

Plants employ various physiological, biochemical, and molecular mechanisms to cope with drought stress, including stomatal closure, osmotic adjustment, and activation of stress-responsive genes. Key mechanisms also include the production of protective proteins, calcium signaling, and the abscisic acid (ABA) biosynthesis pathway (Jafari et al., 2019). A comprehensive understanding of the molecular mechanisms regulating pistachio development and stress responses is essential for improving its productivity and resilience. In this context, the genes encoding zeaxanthin epoxidase (ZEP), dehydrin (DHN) and calcium-dependent protein kinases (CDPK), are of particular interest due to their critical roles in plant stress tolerance and physiological regulation.

Zeaxanthin epoxidase (ZEP) is an essential enzyme in the biosynthesis of abscisic acid (ABA), a phytohormone that regulates critical processes such as seed dormancy, germination, and response to environmental stressors. Its role in ABA biosynthesis highlights its importance in managing water stress and maintaining plant homeostasis (Park et al., 2008; Liang et al., 2022). Dehydrins (DHNs) are a group of late embryogenesis abundant (LEA) proteins that are critical for enhancing plant tolerance to abiotic stress factors, particularly dehydration and cold stress. They are believed to stabilize cellular structures and protect macromolecules from damage induced by stress (Amara et al., 2014; Falavigna et al., 2015; Yu et al., 2018). Calcium-dependent protein kinases (CDPKs) are an essential component of the plant calcium signaling network. CDPKs play a central role in the plant calcium signaling network, mediating responses to various biotic and abiotic stimuli by transducing calcium signals into specific cellular responses. They are involved in numerous physiological processes, including growth, development, and environmental adaptation (Bansal et al., 2016). Understanding the functions and interactions of these genes offers insights into the mechanisms of drought tolerance and provides a foundation for developing strategies to improve pistachio resilience to water scarcity.

Using drought-resistant rootstocks is a key strategy for sustainable pistachio production in water-limited environments. Most pistachio orchards in Iran are grafted onto the Badami-Zarand rootstock, which faces challenges due to its low growth rate and susceptibility to certain diseases. UCB1, a hybrid pistachio rootstock developed at the University of California, Berkeley, has shown potential, although its compatibility with Iranian pistachio cultivars, the most diverse in the world, remains unclear (Ferguson et al., 2005). Given that the use of *P. atlantica and P. integerrima* has become widespread worldwide in recent decades due to their drought and salinity tolerance, faster growth and higher yield and disease resistance), we selected these hybrids as promising candidates for drought tolerance (Akbari et al., 2020). However, the tolerance of these rootstocks to abiotic stresses, particularly drought, must be thoroughly evaluated before their widespread adoption in pistachio orchards.

While the scion variety influences fruit properties, the choice of rootstock plays a critical role in the plant’s adaptation to water stress. Although the impact of roots on rootstock behavior can be empirically measured, the underlying mechanisms driving these effects remain poorly understood.

In this study, we used nine-month-old saplings from seven clonal interspecies hybrids *of Pistacia atlantica* × *Pistacia integerrima*, named and encoded as Arota, C2, C16-1, C8-3, C4-2, C9-4 and UCB1, with the aim of investigating the morphological, physiological, and gene expression changes in the drought-sensitive ‘C4-2’ and drought-resistant ‘C9-4’ clones that have shown more promise to identify more resilient pistachio rootstocks capable of thriving in water-limited environments.

## 2 Materials and methods

### 2.1 Plant material and experimental set-up

This study was carried out in June 2023 in a greenhouse at the Faculty of Agricultural Technology, University of Tehran. For the experiments, nine-month saplings of seven clonal interspecies hybrids of *Pistacia atlantica* × *Pistacia integerrima* and the UCB1 were used. These hybrids were selected from among 222 controlled crosses based on the uniformity for their relative height and diameter growth (Akbari et al., 2020), and micropropagated at the Royeshe Sabze Farda Research Center (Pistat). The plant material was transplanted into 10 L pots containing 50% soil and 50% perlite + sand. The pots were arranged according to a randomized complete block design (RCBD) with three replications in each treatment.

Plants were grown in a greenhouse for four weeks to allow the canopy and root system to develop. All plants were fertilized weekly with full-strength Hoagland’s nutrient solution (DaCosta and Huang, 2006) to provide essential nutrients and establish the plants priors to the application of treatments. Soil field capacity (FC), defined as the volumetric water content in the upper part of the soil profile after it becomes fully saturated and drains freely over a 48-hour period without evaporation or rainfall, was determined experimentally using a pressure plate apparatus. The water content at field was determined according to the protocol described by Ottoni Filho et al. (2014). Specific volumetric water content of 15% (V/V) was obtained for the soil under the experimental conditions.

The plants of each clonal hybrid were divided into two groups: control (regular irrigation) and drought stress (Withholding irrigation). The control was regularly irrigated to reach to the field capacity (FC=15%V/V, 1.305 L per pot) every three days during the experimental period (30 days, irrigation=10 times, total water per pot=13.05 L). The second group was subjected to a drought stress by withholding irrigation (Arab et al., 2020). The greenhouse temperature was kept 28/20°C day/night with a relative humidity of 40-60% and 16/8 hours light/dark (500–650 mol m^−2^ s^−1^ flux density) during the experiment. The stress lasted for 30 days, which was the period required for the sensitive plants to show significant signs of water stress, including a strong loss of turgor and the wilting and discoloration of most leaves. At the end of the experiment, the most sensitive and resistant clones were selected based on the leaf relative water content, cell membrane stability index, leaf count, percentage of damaged leaves, vitality and total chlorophyll (T Chl) (data not shown). Subsequently, the morpho-physiological and molecular mechanisms involved in drought stress tolerance were investigated at root system level as follows.

### 2.2 Experimental design

The experiment was arranged as a randomized complete block design (RCBD) with two factors, irrigation (control and withholding irrigation) and clonal hybrids with three biological replicates (7 clonal hybrids x 3 replicates x 2 treatments). Analysis of variance (ANOVA) was conducted with R software (R 4.3.2) using the “car”, “ggplots”, “emmeans”, “easyanova”, “DescTools” and “sjstats” packages. Means were compared using Duncan’s Multiple Range Test (P<0.05). The normality of each trait was tested using the Shapiro-Wilk approach.

### 2.3 Measurements

#### 2.3.1 Growth parameters

##### 2.3.1.1 Root fresh and dry weight

The fresh and dry weights of the roots were measured using a scale with a precision of ±0.1 mg. The dry weight of the plant fractions was determined after drying them in an oven at 85°C for 72 hours (Zarehaghi et al., 2017).

##### 2.3.1.2 Root architecture

At the end of the experiment, the roots were gently removed from the pots, separated from the crown and photographed by using a RGB digital camera. Image processing was carried out using the GiaRoots^®^ software (Galkovskyi et al., 2012), collecting the following parameters: Average Root Width (AVRW: the mean value of the root width estimation computed for all pixels of the medial axis of the entire root system), Network Area (NWCA: area of the convex hull that encompasses the image), Network Width (NWWI: the number of pixels in the horizontal direction from the left-most network pixel to the right-most network pixel.), Network Depth (NWDP: number of pixels in the vertical direction from the upper-most network pixel to the lower-most network pixel), Network Length Distribution (NWLD: fraction of network pixels found in the lower 2/3 of the network which is defined based on network depth), Major Ellipse Axis (MAEA: the length of the major axis of the best fitting ellipse to the network), Network Volume (NWVL: sum of the local volume at each pixel of the network skeleton, as approximated by a tubular shape whose radius is estimated from the image), Network Width to Depth Ratio (NWWD: The value of network width divided by the value of network depth).

#### 2.3.2 Physiological and biochemical parameters

##### 2.3.2.1 Glycine betaine concentration

A total of 0.5 g of dry root was placed in an Erlenmeyer flask containing 25 ml of distilled water. The flask was shaken for 48 hours at a temperature of 25°C. Then, 1 ml of the extraction solution was mixed with 2 N sulfuric acid and 0.5 ml of the resulting mixture was transferred into a test tube and placed in a cold-water bath for 1 hour. A 0.2 ml of potassium iodide solution (prepared from 7.15 g iodide and 20 g potassium iodide) was added to the test tube, and the tube was stored at 4°C for 14 hours. The samples were then centrifuged for 15 minutes at 10,000 rpm using a refrigerated centrifuge. The granular crystals were dissolved in 9 ml of dichloromethane and shaken for 2 hours. Afterward, samples absorbance was measured at 365 nm using a spectrophotometer (HITACHI U-1900 UV-Visible spectrophotometer). The concentration of glycine betaine was determined using a standard curve, and the results were expressed in µmol g^−1^ dry weight (Di Martino et al., 2003).

##### 2.3.2.2 Proline concentration

The procedure involved the use of a solution of acid-ninhydrin prepared by heating 1.25 g ninhydrin in 30 ml glacial acetic acid and 20 ml 6 M phosphoric acid until fully dissolved and kept at 4°C before using within 24 hours. Roots from greenhouse-grown pistachio saplings were sampled and approximately 0.5 gram were homogenized in 10 ml of 3% sulfosalicylic acid solution. The resulting homogenate was filtered through Whatman filter paper and 2 ml of the filtrate were mixed with 2 ml of acid-ninhydrin solution and 2 ml of glacial acetic acid. The mixture was then heated at 100°C for 1 hour and then cooled in an ice bath to end the reaction. The reaction mixture was extracted with 4 ml of toluene by vigorously stirring the test tube for 15-20 seconds. The toluene phase containing the chromophore was separated from the aqueous phase at room temperature and the absorbance was measured at 520 nm using toluene as a blank. The proline concentration was determined by using a standard curve and calculated on the basis of the fresh weight of the sample (Irigoyen et al., 1992).

##### 2.3.2.3 Total soluble carbohydrate content (TSC)

Total soluble carbohydrates (TSC) were determined by analyzing 95% ethanol extracts from root tissues. For each sample, 0.5 g of freshly harvested roots were finely crushed in 5 ml of 95% (v/v) ethanol. To remove the insoluble fraction, the extract was washed twice with 5 ml of 70% ethanol. All the soluble fractions were then centrifuged at 3500 g for 10 minutes, the supernatants collected and carefully stored at 4°C. For the determination of TSC, 0.1 ml of the alcoholic extract was added to 3 ml of a freshly prepared anthrone solution (containing 150 mg anthrone and 100 ml of 72% (w/w) H_2_SO_4_) and the mixture was heated in a boiling water bath for 10 minutes. After cooling, the absorbance was measured at 625 nm to assess the TSC content (Irigoyen et al., 1992).

##### 2.3.2.4 Hydrogen peroxide (H_2_O_2_)

The lyophilized root tissue was crushed and mixed with 0.1% cold trichloroacetic acid (TCA) to measure the H_2_O_2_ concentration. The mixture was then centrifuged at 12,000 × g for 15 min in a refrigerated centrifuge. After centrifugation, 10 mM phosphate buffer (pH 7.0) and 1 M iodate potassium (KI) solution were added to 0.5 mL of the supernatant. The absorbance was then measured at 390 nm. A standard curve with known concentrations of H_2_O_2_ was established to determine the amount of hydrogen peroxide (Ben Abdallah et al., 2017).

##### 2.3.2.5 Malondialdehyde concentration (MDA)

To perform the MDA assay, fresh roots weighing 0.25 g each were ground separately in 5 ml of 1% trichloroacetic acid (TCA). The resulting mixture was centrifuged at 5000 rpm in a refrigerated centrifuge for 10 minutes. Subsequently, 4 ml of 20% TCA containing 0.5% thiobarbituric acid was added to 1 ml of the supernatant. The mixture was heated to 95°C for 30 minutes, then cooled rapidly in an ice bath, and the absorbance was measured at 450 nm, 532 nm, and 600 nm using a spectrophotometer. The MDA concentration was determined using the following equation (Zhao et al., 1993).


$$\text{MDA}\left({\text{µmol g}}^{-1}\text{FW}\right)=6.45\left(\text{OD}532-\text{OD}600\right)–\;0.56\text{OD}450$$

#### 2.3.3 Enzyme assays

##### 2.3.3.1 Enzyme extraction

A volume of 5 ml of extraction buffer, comprised of 50 mM K-phosphate buffer and 0.1 mM Na_2_-EDTA with a pH of 7.6, was utilized for extracting enzymes from 0.5 g of leaf tissue using a mortar and pestle. Following centrifugation of the homogenate at 16,000×g for 20 min, the resulting supernatant was employed for assessing various enzymes. It is important to note that all stages involved in the preparation of enzyme extracts were carried out at a temperature of 3°C.

##### 2.3.3.2 Catalase activity (CAT)

A 0.1 g root sample was mixed with 3 ml of a reaction buffer consisting of standard hydrogen peroxide and 50 mM potassium phosphate buffer with a pH of 7.8, and 200 μl of the extraction enzyme. The maximum absorption of peroxide occurs at 240 nm, and in the presence of CAT, hydrogen peroxide is degraded, resulting in a decrease in its absorbance at 240 nm due to the removal of oxygenated water (MacRae and Ferguson, 1985).

##### 2.3.3.3 Guaiacol peroxidase (GPX)

For the assay, 50 μL of the enzyme extract was combined with 100 mM phosphate buffer (pH 7.0), 0.1 μM EDTA, 5.0 mM guaiacol and 15.0 mM H_2_O_2_ resulting in a final volume of 2.0 mL. The absorbance at 470 nm was measured both immediately after the addition of H_2_O_2_ and again after 1 minute. This allowed the oxidation of guaiacol to be observed, leading to the formation of tetraguaiacol. To determine the enzyme activity, the absorbance difference (ΔA470) was divided by the molar extinction coefficient of tetraguaiacol, which was calculated to be 26.6 mM^−1^ cm^−1^. The enzyme activity was expressed as μmol of reduced H_2_O_2_ per minute per milligram of protein. Based on this calculation, it was determined that 4.0 moles of H_2_O_2_ are reduced to generate 1.0 mol of tetraguaiacol (Pakzad et al., 2019).

#### 2.3.4 FTIR-ATR analysis

FTIR-ATR (Fourier Transformed Infrared-Attenuated Total Reflectance Spectroscopy) spectra of dried and grinded samples (leaves and roots of control, water-stressed and recovered plants) were recorded using a Perkin Elmer Spectrum Two spectrophotometer equipped with a 2 × 2 mm diamond crystal (4000–400 cm^−1^ range with a 2 cm^−1^ resolution).

#### 2.3.5 Gene expression analyses

##### 2.3.5.1 RNA extraction

For the RNA extraction, the most resistant and sensitive clones were selected among the clones exposed to drought for 30 days (2 clonal hybrids x 3 replicates x 2 treatments=12 total samples). Total RNA was extracted according to the Moazzam Jazi et al. (2015) modified protocol. 100 mg of roots was added to 100 μl of the cetyltrimethylammonium bromide (CTAB), 50 μl polyvinylpyrrolidone (pvp) 20% and 35 μl of β- mercaptoethanol. The mixture was incubated at 60°C for 15 min, then 350 μl of the RB lysis buffer was added. The mixture was incubated at 60°C for 10 min and then clarified by centrifugation at 14,000 x g for 5 min at 4°C. The supernatant was then removed and transferred to another tube and 200 μl of chloroform was added. The mixture was incubated at room temperature for 3 min and centrifuged again. The supernatant was collected again and 250 μl ethanol 96% was added. The supernatant was transferred to the collection tube and the columns were washed with RW buffer. The RNA was eluted in 50 μl RNase free water and stored at −80°C. The quantity and quality of the extracted RNA was determined using the Nanodrop ND-1000 spectrophotometer.

##### 2.3.5.2 qRT-PCRs

Quantitative real-time polymerase chain reactions (qRT-PCRs) were performed on three genes associated with drought stress: *ZEP* for zeaxanthin epoxidase, *CDPK*s for Ca^2+-^dependent protein kinases and dehydrin. The primer pairs utilized are listed in **Table 1**. Reverse transcription of RNA samples was carried out using the iScript™ gDNA Clear cDNA Synthesis Kit (Bio-Rad, Hercules, CA, USA), following the manufacturer’s guidelines. qRT-PCRs reactions were performed using the SsoAdvanced Universal SYBR^®^ Green Supermix (Bio-Rad) and the CFX96 Touch Real-Time PCR Detection System (Bio-Rad). The thermal cycling conditions were as follows: an initial denaturation at 95°C for 3 minutes, followed by 40 cycles of 95°C for 10 seconds and 57°C for 30 seconds. The specificity of amplification for each primer pair was confirmed by assessing the melting curve, with an increment of 0.2°C every 5 seconds from 65 to 95°C. Each qRT-PCRs assay was performed in triplicate with three biological replicates. Elongation factor 1α (*EF1α*) was selected as the reference gene for normalization, and the comparative Ct method (2^−ΔCt^ method) was used to analyze the expression levels of the target genes.

**Table 1:** Primer sequences used for qRT-PCRs analysis.

Reference	Primer name	Sequences (5’-3’)
Moazzzam Jazi et al., 2017	F-CDPK	CATGGCCCAACATATCAGACAG
	R-CDPK	CCACAATCCAGGGGTGACATA
	F- DHN	GCGAGCAGAAAGGGCTGTA
	R-DHN	TGCTGCTTCACGCCATCAC
	F-ZEP	ACTTTGCAACAAATCCTCGCT
	R-ZEP	CCTCACCTTCGACCATATTCCAT
	F-EF1α	GGCAAGGTATGATGAAATCGTG
	R-EF1α	ATCACCCTCAAATCCAGAGATG

## 3 Results

### 3.1 Growth parameters

#### 3.1.1 Root characteristics

**Figure 1:**
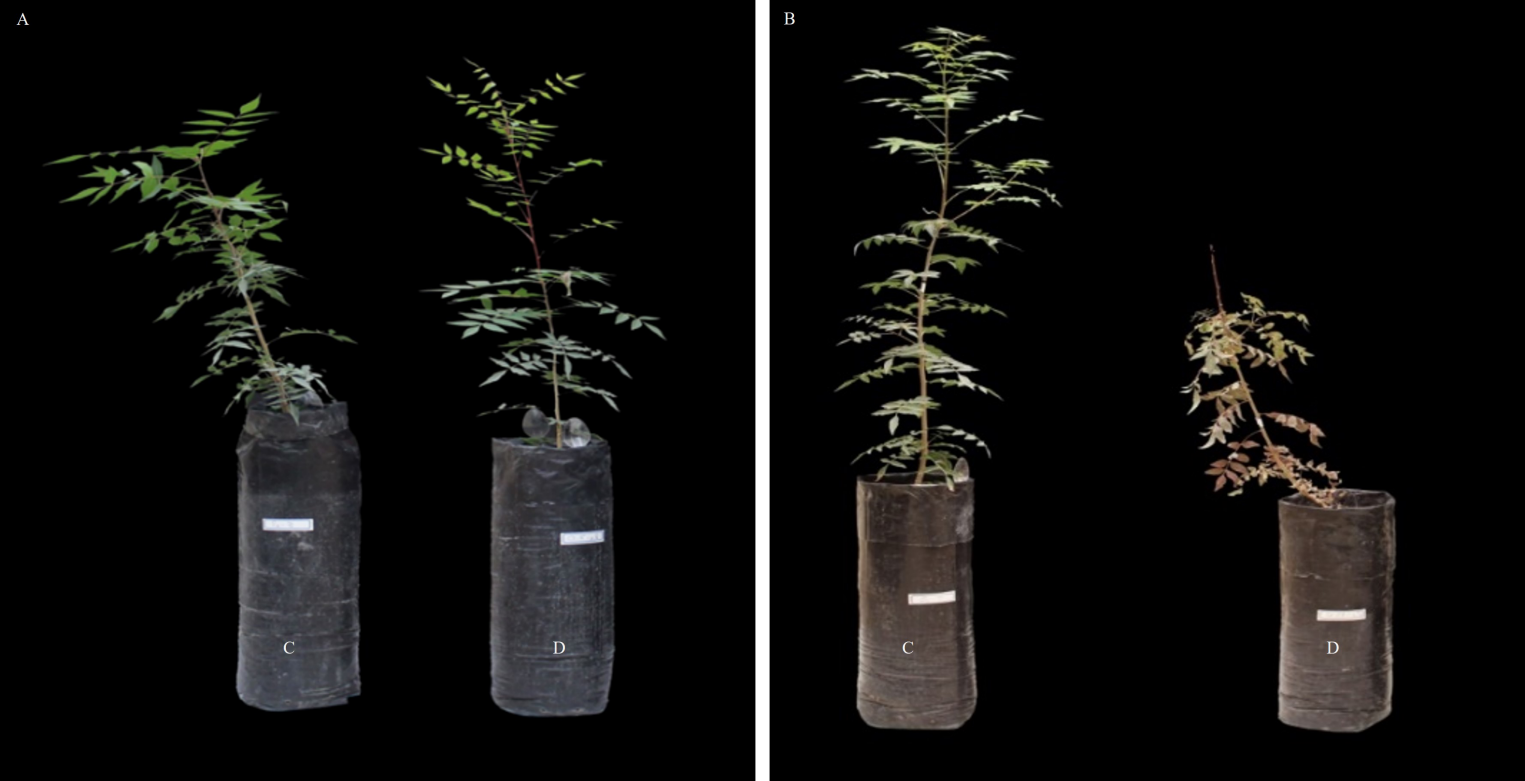
Effect of water stress on the resistant (**A**, C9-4) and sensitive (**B**, C4-2) clones. [C, Control; D, Drought].

**Figure 2:**
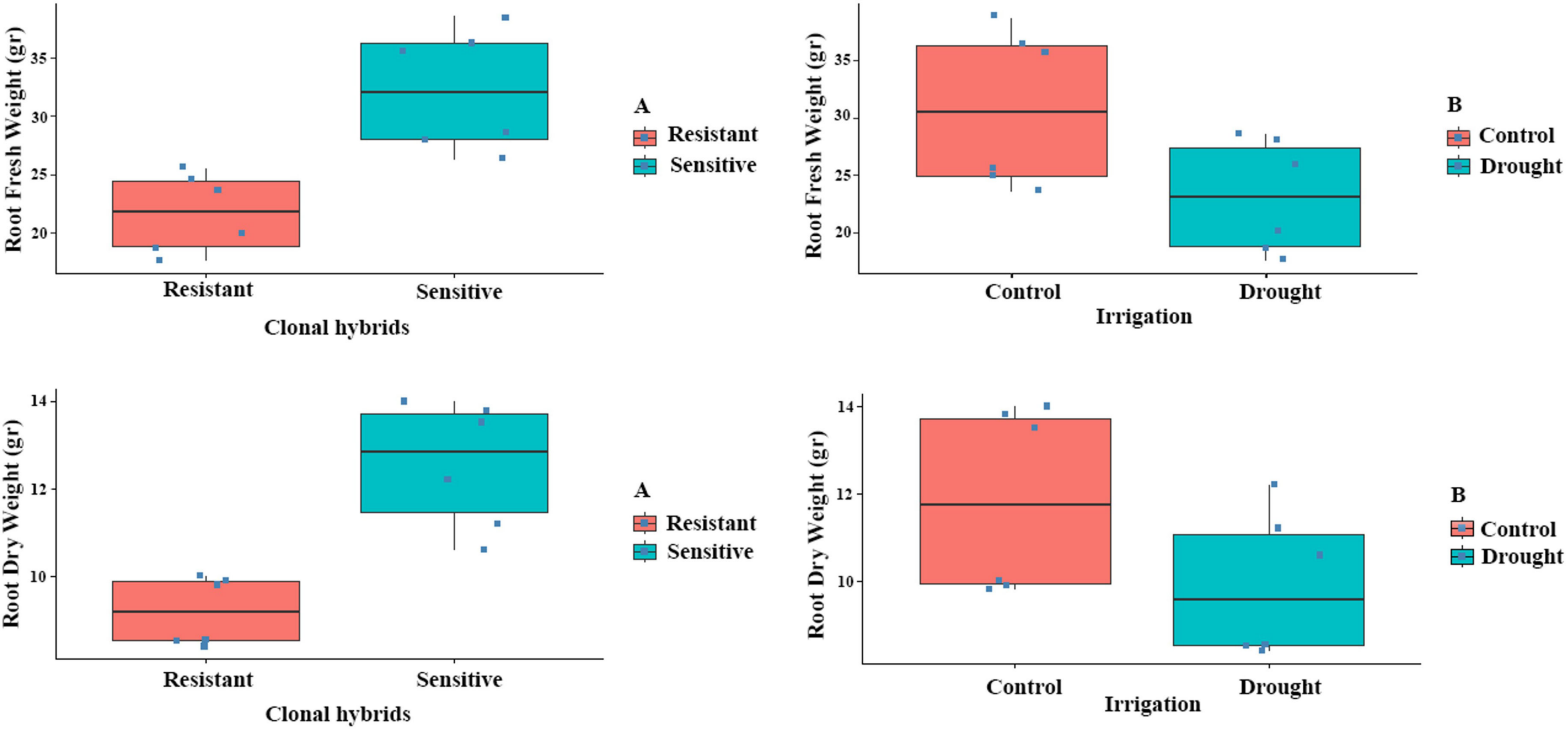
Effect of clonal hybrids and irrigation on RFW and RDW of resistant and sensitive clones. Bars indicate standard error.

After the completion of the experiment, the most resistant and sensitive clones were selected. The results showed that C9-4 was the most resistant clone, as it had the highest leaf relative water content, cell membrane stability index, T Chl, vitality and the lowest percentage of damaged leaves under water stress (**Figure 1A**). On the contrary, C4-2 was the most sensitive clone to drought stress as it had the lowest leaf relative water content, cell membrane stability index, vitality and T Chl, and the highest percentage of damaged leaves under water stress (**Figure 1B**). Root growth was determined based on root fresh weight (RFW) and root dry weight (RDW). RFW and RDW showed significant variation by the effects of clonal hybrids and irrigation (*P ≤ 0.01*) and non-significant by the interaction (**Supplementary Table S1**). As expected, the highest values for RFW and RDW were recorded under control conditions and in the sensitive clones compared to the resistant ones (**Figure 2**). The higher root biomass in resistant clones (C9-4) under control conditions likely reflects their genetic predisposition for more extensive root growth. Although significant morphological changes in root systems generally require prolonged stress exposure.

#### 3.1.2 Root architecture

**Figure 3:**
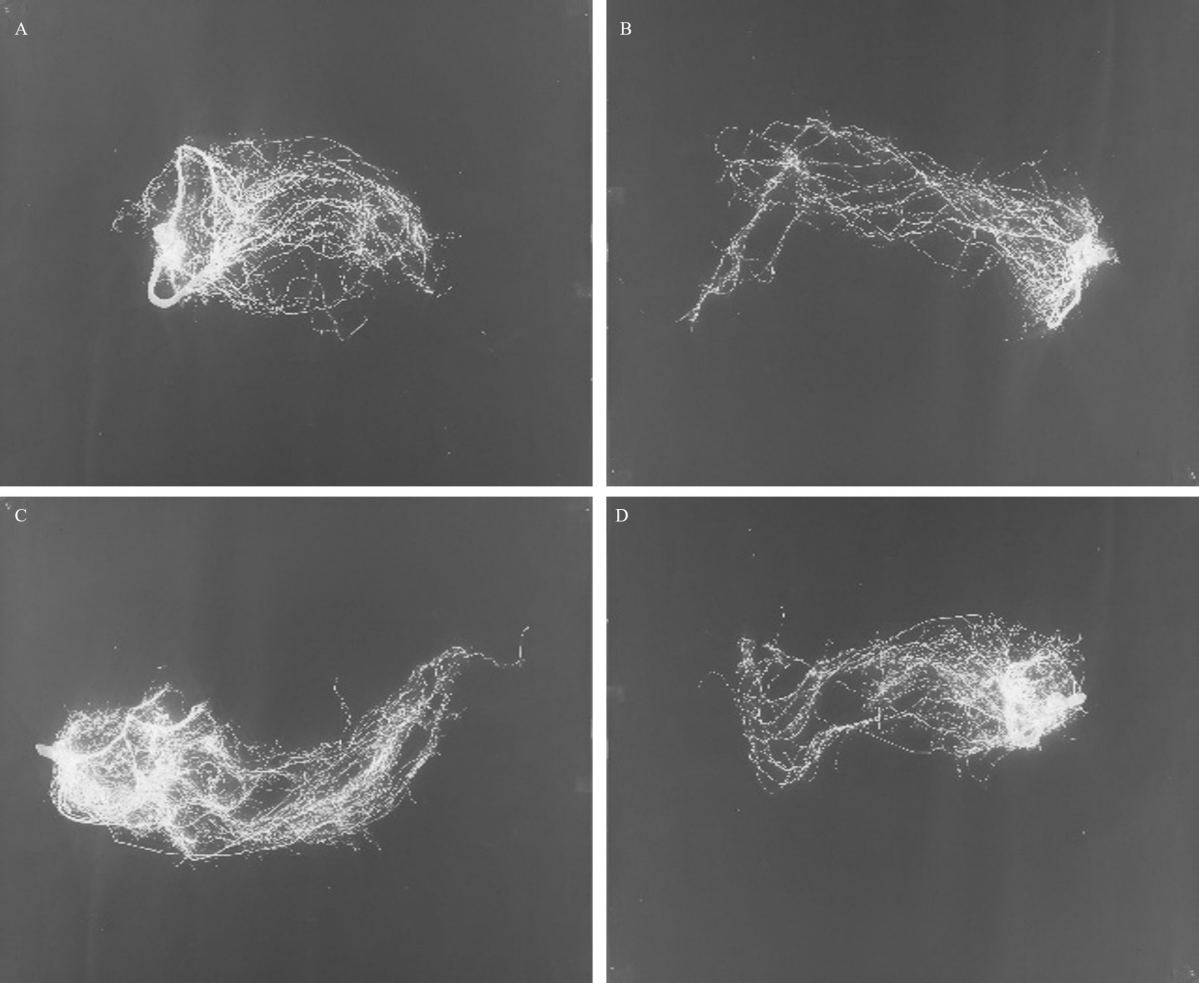
Roots of pistachio clonal hybrids under control [**A** (Resistant), **C** (Sensitive)] and water stress condition [**B** (Resistant), **D** (Sensitive)].

Imaging of the ‘pistachio’ roots 30 days after the start of the experiment showed that water withholding caused changes in the root architecture in the resistant clone. The analysis of variance showed a significant difference by the effects of the irrigation for AVRW and NWVL (*P ≤ 0.01*), of the clonal hybrids for AVRW, NWCA, NWDP (*P ≤ 0.01*) and of interaction of clonal hybrids with irrigation for AVRW, NWCA (*P ≤ 0.01*) and NWVL (*P ≤ 0.05*). This is despite the fact that none of the effects were significant for NWWI, NWLD and NWWD (**Table 2**). The highest AVRW and NWCA were measured in the resistant clones in the control. Water withholding reduced these two parameters in the resistant clones while no significant difference were recorded in sensitive clones for these two traits between control and drought stress treatment. The highest NWVL was observed in sensitive clones. Drought treatment reduced NWVL in the resistant clones but had no effect on the sensitive clone. The drought treatment increased NWDP in the resistant clones and these clones had the highest value of this parameter under the drought treatment compared to other treatments (**Table 2**). In general, the result showed that the root architecture parameters were not affected by water withholding in the sensitive plants (**Figures 3C, D**) while in resistant plants under the drought, average root width, network area and network volume decreased and network depth increased (**Figures 3A, B**).

**Table 2:** Mean values of AVRW, NWCA, NWWI, NWDP, NWLD, MAEA, NWVL and NWWD in different irrigation treatments (I) for two pistachio clonal hybrids (C).

Clonal hybrid	Irrigation		AVRW (cm)	NWCA (cm^2^)	NWWI (cm)	NWDP (cm)	NWLD	MAEA (cm)	NWVL (cm^3^)	NWWD
Resistant	Control	Mean value	0.18^a^	917.5^a^	85.5^a^	71^b^	0.11^a^	107.5^a^	87^b^	1.41^a^
	Drought		0.13^b^	489.3^b^	80.4^a^	78.5^a^	0.12^a^	108^a^	70^c^	1.38^a^
Sensitive	Control		0.11^b^	804.6^a^	87.4^a^	71.3^b^	0.10^a^	107^a^	107^a^	1.45^a^
	Drought		0.11^b^	816.5^a^	81.3^a^	72^b^	0.11^a^	108.2^a^	108^a^	1.43^a^
Resistant	Control	STDEV	0.005	34.13	0.66	0.78	0.005	1.52	2.57	0.30
	Drought		0.004	19.10	0.44	0.44	0.003	1.33	1.32	0.040
Sensitive	Control		0.001	22.92	0.30	0.50	0.01	0.51	4.95	0.015
	Drought		0.002	32.91	2.57	0.66	0.004	1.36	4.21	0.013
ANOVA	C		**	ns	ns	ns	ns	ns	**	ns
	I		**	**	ns	**	ns	ns	ns	ns
	C*I		**	**	ns	ns	ns	ns	*	ns

Within each column in every hybrid, means superscript with different letters are significantly different by Duncan’s range test (P< 0.05). *, **, ns: Significantly differences at of 5% and 1% of probability levels and non-significantly differences, respectively. STDEV is Standard Deviation.

### 3.2 Physiological and biochemical parameters

#### 3.2.1 Glycine betaine, proline and TSC concentration

**Figure 4:**
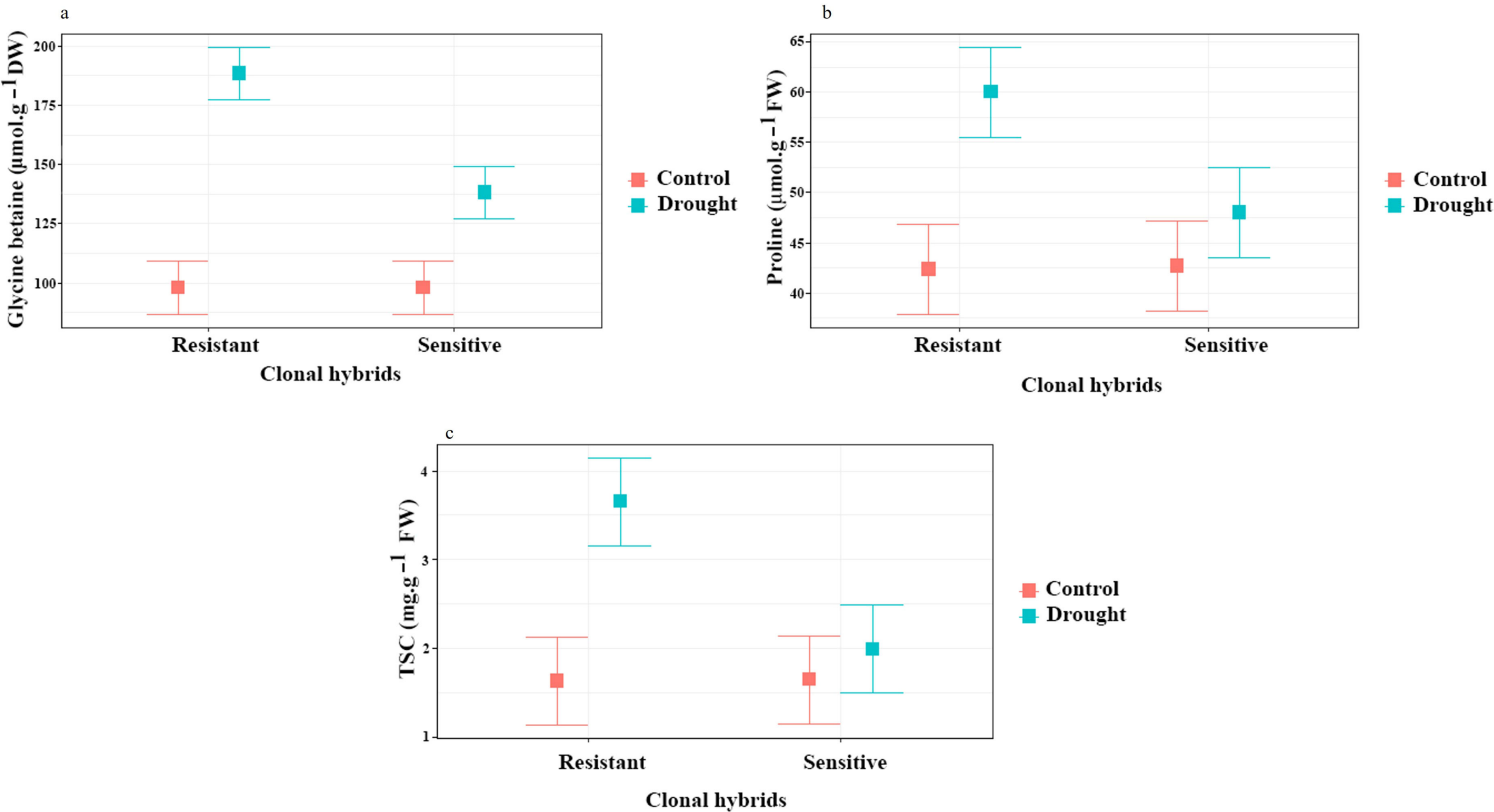
Influence of water stress on glycine betaine **(a)**, proline **(b)** and TSC **(c)** content in roots of two clonal hybrids of pistachio. Bars indicate standard error.

For glycine betaine and proline content, a significant difference was found for clones, irrigation and interaction clones x irrigation (*P ≤ 0.01*). Glycine betaine and proline content significantly increased in response to water stress treatment in resistant and sensitive. Under drought conditions the Glycine betaine content increased by 90% in the resistant rootstock and by 40% in the sensitive rootstock (**Figure 4a**), while proline increased by 42% and 14% in the resistant and sensitive rootstock, respectively (**Figure 4b**). At the 1% level, ANOVA showed a significant difference in root TSC for clonal hybrids, irrigation and interaction of them. TSC content increased by 95% in the resistant rootstock and by 20% in the sensitive rootstock in response to water stress treatment. The sensitive clone had no significant difference to the control in this trait (**Figure 4c**). These increases in osmoprotectant levels likely helped maintain osmotic balance in both clone types under drought stress.

#### 3.2.2 Antioxidant activity (GPX and CAT activity)

**Figure 5:**
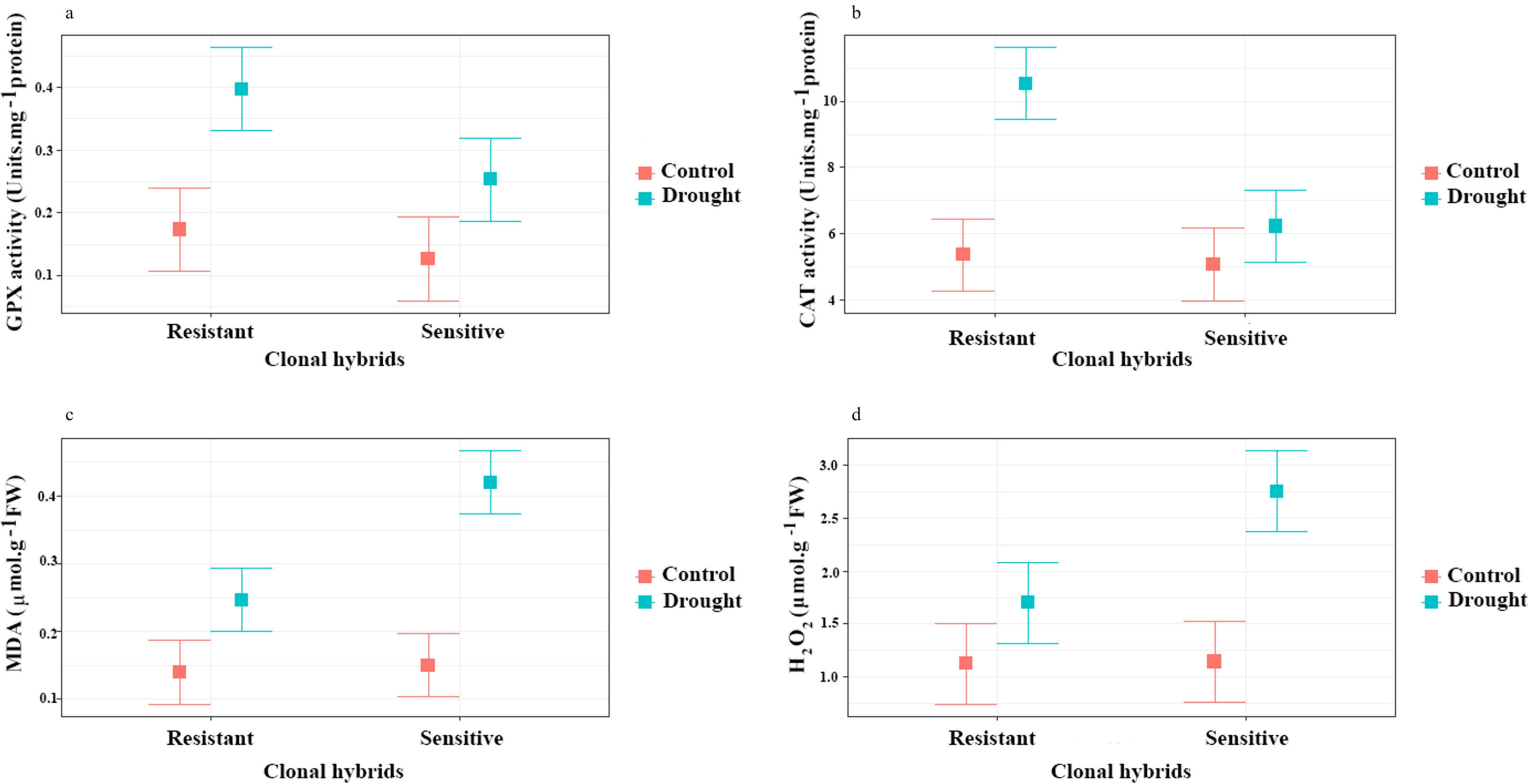
Influence of water stress on GPX **(a)**, CAT **(b)**, MDA **(c)** and d) H_2_O_2_ **(d)** in roots of two clonal hybrids of pistachio. Bars indicate standard error.

For GPX and CAT content, a significant difference was observed between hybrids and irrigation (*P ≤ 0.01*) and interaction of clonal hybrids with irrigation respectively (*P ≤ 0.05, P ≤0.01*). As shown in **Figure 5**, GPX and CAT enzymes activities increased (2.5 and 2 times, respectively) by induction of drought treatment compared to normal environment in resistant clone, but there is no significant difference between controls and sensitive clones that were exposed to drought. The results of this research show that sensitive hybrids were not able increase antioxidant activity. Sensitive clones failed to significantly increase antioxidant activity (GPX and CAT) under drought stress, indicating a reduced capacity to manage oxidative damage. This lack of upregulation in these enzymes likely contributes to their higher susceptibility to oxidative stress, impairing their ability to cope with drought-induced damage.

#### 3.2.3 MDA and H_2_O_2_

Based on the results of variance analysis (**Supplementary Table S1**), MDA and H_2_O_2_ content had a significant difference between clonal hybrids, irrigation and interaction of clonal hybrids with irrigation (*P ≤ 0.01*). Contents of MDA and H_2_O_2_ increased in response to the drought treatment compared to the control condition. The lowest destructive effects of drought on lipid peroxidation were related to the control plants. Considering the results of this research, induction of drought treatment induced the content of MDA (**Figure 5c**) and H_2_O_2_ (**Figure 5d**) in both sensitive and resistant clones but in the sensitive clone, this induction was more than resistant rootstock.

### 3.3 FTIR-ATR

**Figure 6:**
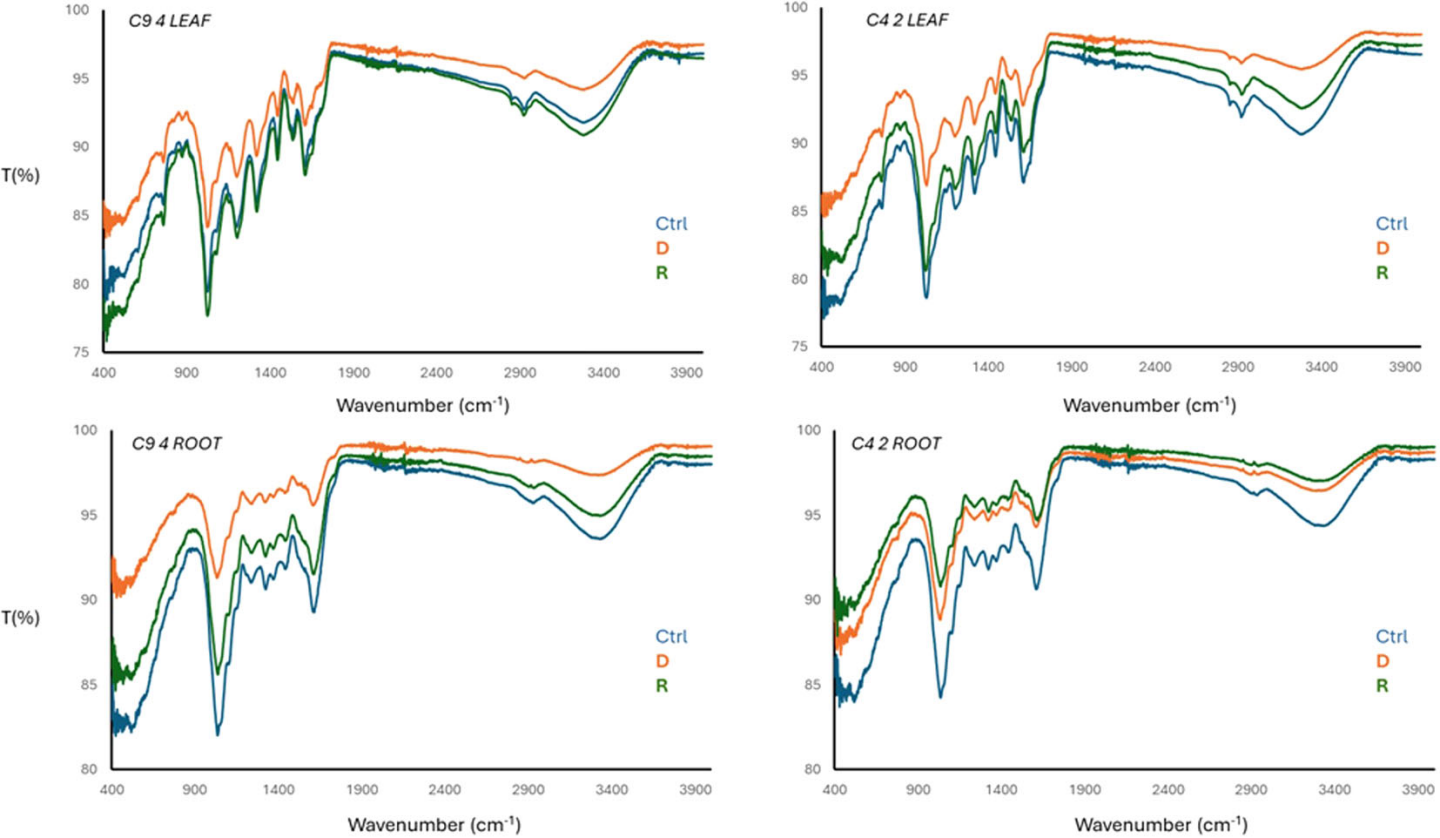
FTIR-ATR spectra of C9-4 (resistant) and C4-2 (sensitive) samples of dried and grinded samples leaves and roots. Control (Ctrl), Drought (D) and Recovery (R).

In the perspective of the investigation on the samples presented in this paper (leaves and roots of control, drought and recovery stage for resistant and sensitive clones), Transformed Infrared Spectroscopy in Attenuated Total Reflectance mode (FTIR-ATR) was exploited to study the different response to dehydration stress and related, subsequent plant recovery. Plants with no irrigation treatment received water in a similar quantity as control plants, and 30 days after recovery samples were collected for FTIR analysis, which was carried out on samples dried and grinded. According to (Ertani et al. (2018) (**Figure 6**), aliphatic and -OH signal decrease to water stress (dehydration, D) and increase after recovery (R), with respect to a control sample (Ctrl). However, -OH signal is not only related to the water bulk at surface, interphase or entrapped phase, but it also belongs to phenolic components normally produced to switch an antioxidant response to stress conditions and to compose the lignin skeleton of the leaves and the roots. A slight modification in -NH bending moiety was also found as associated with water stress conditions.

### 3.4 Gene expression analysis

Further investigation into the mechanisms underlying plant drought resistance involved analyzing the expression of stress-responsive genes. The coordinated expression and regulation of *DHN*, *CDPK*, and *ZEP* genes contribute to the plant’s ability to withstand drought stress. Dehydrins protect cellular components from damage induced by dehydration-, while CDPKs coordinate signaling pathways that regulate gene expression and physiological responses. ZEP enzymes are involved in ABA biosynthesis, which plays a central role in drought stress signaling and adaptation (Xiong and Zhu, 2003; Moazzzam Jazi et al., 2017; Yang et al., 2021).

**Figure 7:**
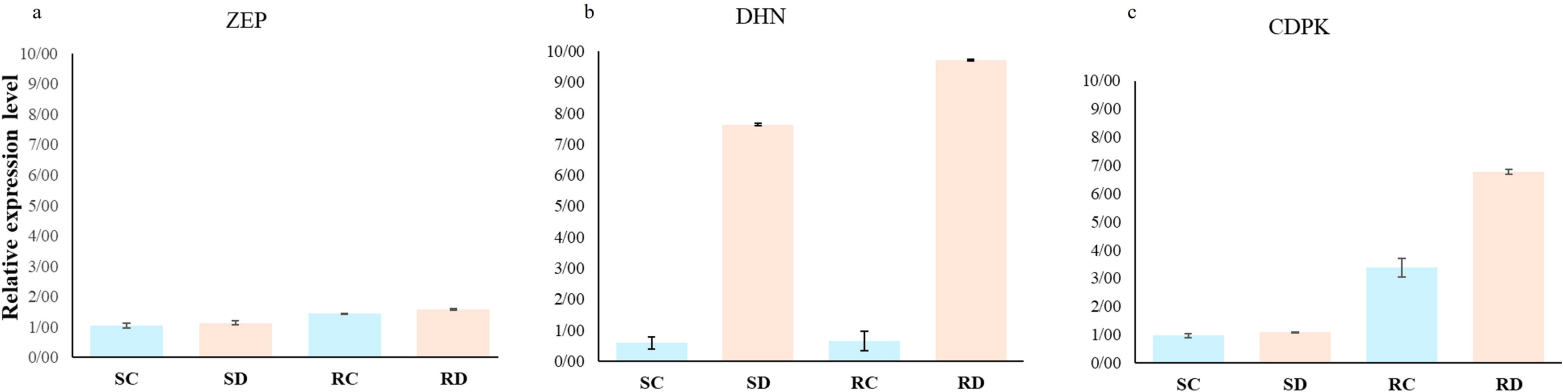
Relative expression levels of the genes ZEP **(a)**, DHN **(b)** and CDPK **(c)** detected in pistachio plants exposed to drought stress detected by qRT-PCRs. EF1α was used as a reference gene. Data are displayed as mean values of the three replicates with standard error. [SC: sensitive control, SD: sensitive drought, RC: resistant control, RD: resistant drought].

*DHN*, *CDPK*, and *ZEP* were selected to perform qRT-PCRs analysis in root samples after 30 days of drought treatment. All primer pairs yielded a solitary band of the expected size on gene electrophoresis, accompanied by a peak on the melting curve. This suggests the dependence on gene expression profiling. Evaluation of these three genes shows an increase in gene expression in the treated plants compared to the control plants. In detail, a small variation in the expression level of the *ZEP* gene was observed in both the sensitive and resistant plants (**Figure 7a**). The *DHN* gene exhibited a twelve-fold and fourteen-fold increase in expression levels under withholding conditions compared to normal conditions in the sensitive and resistant rootstocks, respectively (**Figure 7b**). For the *CDPK* and *DHN* genes, a slight increase was found for the putative-sensitive clone, while in the resistant rootstock, the expression increased seven-fold under drought stress and three-fold under normal conditions (**Figure 7c**).

## 4 Discussion

Plants constantly face various abiotic and biotic stresses in their natural environment, with drought being one of the most detrimental factors affecting plant growth and productivity (Varshney et al., 2013). While pistachio is relatively resistant to drought and salinity, these remain critical factors limiting its growth and productivity in Iran (Pourmohammadali et al., 2019). Root morphology and architecture, in particular, play an important role in drought tolerance, suggesting that using drought-resistant rootstocks could be an effective strategy for sustainable pistachio production in water-limited environments. In this study, pistachio interspecific hybrid rootstocks were preselected as resistant (C9-4) and sensitive (C4-2) clones after a 30-day experiment under limited irrigation. These clones were then subjected to physiological, biochemical, and molecular analyses to examine their responses to drought stress.

Changes in root structure, distribution, and architecture are thought to play a significant role in the drought tolerance of resilient plant species (Yang et al., 2021). Normally, roots allocate over 40% of the carbon utilized by plants. However, under water stress, the biomass allocated to the root system increases, and in drought-prone soils, the root-to-shoot ratio is higher in drought-tolerant plants (Nwaogu, 2014).

Moreover, the drought resistance trait in plants is often associated with a deep root system and specific root architecture (Esmaeilpour et al., 2015). These drought-induced changes enhance a plant’s ability to uptake water within a limited time frame, depending on water availability and environmental factors (Thangthong et al., 2018). However, in our experiments, the expected increase in root biomass was not observed in the resistant clones, suggesting that they might employ alternative drought-tolerance mechanisms. According to Sánchez-Blanco et al. (2014), the root system does not always change as a result of drought stress, and Saadatmand et al. (2007) observed no increase in root biomass in the drought-resistant Pistachio cultivar Sarakhs under water stress. The higher root biomass observed in the resistant clones (C9-4) at baseline can be attributed to their inherent genetic traits, which result in greater root growth even under control conditions. This suggests that these clones are naturally predisposed to develop more extensive root systems, which might contribute to their ability to better withstand stress conditions. Furthermore, according to recent studies, plants typically require longer periods of stress exposure to achieve significant morphological changes in their root systems. For example, Vives-Peris et al. (2020) reported that increased root biomass often occur after prolonged stress exposure. Additionally, some of researchers observed that during short-term drought stress, plants prioritize physiological adaptations, such as adjusting metabolic processes, rather than undergoing rapid morphological changes (Bhargava and Sawant, 2013). In our experiment, water withholding was applied for a duration of 30 days. Given this relatively short period, it is likely that our saplings did not have enough time to undergo substantial morphological changes in their root systems. Instead, they likely responded to the stress by utilizing physiological adaptations, such as altered metabolic processes, to cope with the limited water availability. However, while increased investment in roots is crucial for survival, it must be optimized through a strategic allocation of roots in space and time (root architecture) (Nwaogu, 2014). Root system architecture (RSA) refers to the spatial arrangement of roots within the soil, including their connectivity and distribution. This arrangement is shaped by growth rates, branching frequency, and the gravitational orientation of different root types (Larue et al., 2022). Recent advances in “omics” approaches have greatly improved our understanding of the regulatory mechanisms involved in remodeling RSA under drought conditions and identifying the genes and other regulatory elements involved (Ranjan et al., 2022). Plants exposed to low-moisture conditions tend to develop broader root systems and larger root diameters. In general, plants under water stress tend to produce thinner roots compared to non-stressed plants. Furthermore, under deficit irrigation, the reduction in root volume (NWVL) often exceeds the decrease in dry weight, leading to an increase in root density (Sánchez-Blanco et al., 2014). Our experimental results revealed that the sensitive clone did not exhibit significant changes in root architecture under drought, showing no differences compared to the control. In contrast, the resistant clone was affected by water withholding, showing a decrease in AVRW, NWCA, and NWVL, while NWDP increased. These findings align with those of Sadat-Hosseini et al. (2019), who reported that NWCA and NWVL decreased in Chandler walnut under drought, while AVRW and NWDP showed no significant difference from the control. Previous studies have demonstrated that *Pistacia atlantica* possesses a dense network of deep roots capable of extracting water from deeper soil reducing the number and branching of surface roots while increasing the growth of deep roots (Nwaogu, 2014). This shift enhances its ability to access and store moisture in deeper soil layers, preventing evaporative loss from roots (Limane et al., 2014).

Plants accumulate significant amounts of low-molecular-weight organic solutes to resist drought stress. Among them soluble sugars, proline, and other amino acids, which help regulate the osmotic potential of cells and improve water uptake (Khoyerdi et al., 2016). During hydric stress, proteins are hydrolyzed by amino acid-releasing proteases, either for storage or for osmotic regulation. In pistachio, sucrose, glycine betaine, and proline act as compatible solutes, facilitating drought adaptation. The synthesis and accumulation of compatible solutes is a common mechanism in plants for osmotic adjustment, primarily serving to retain water within cells without interfering with normal metabolism (Di Martino et al., 2003). Biochemical changes recorded in our experiment showed an increase in glycine betaine and proline contents in the roots of both resistant and sensitive plants during water stress, with much higher concentrations in the resistant clone. Moreover, the accumulation of glycine betaine was three times higher compared with proline.

In the analyzed samples, the TSC content in roots increased during water stress only in the resistant clone. These results align with previous studies reported by Ryan and Ash (1996) and Khoyerdi et al. (2016).

The formation of ROS species is common in plants experiencing drought stress, these contribute to lipid peroxidation, protein degradation and nucleic acid damage. To mitigate the harmful effects of ROS, plants have developed an antioxidant defense system that includes enzymes such as GPX, SOD, CAT, and others (Bano et al., 2012). The samples exposed to drought stress showed increased GPX and CAT enzyme activity, which was significant in the resistant clone. Similar results were found in UCB-1 pistachio rootstock (Pakzad et al., 2019), where GPX, CAT and APX increased under drought, whether alone or combined with salicylic acid application in *Pistacia vera* seedlings (Reyhani Haghighi et al., 2021). The increased activity of GPX and CAT suggests their crucial role in scavenging ROS and protecting plant cells from oxidative damage during drought stress. The antioxidant defense system helps prevent cellular damage by neutralizing ROS, thereby maintaining cellular integrity and promoting stress tolerance. This link between antioxidant activity and the protective role against drought-induced oxidative stress highlights the importance of these enzymes in mitigating the harmful effects of drought on plant health (Laxa et al., 2019). The failure of sensitive clones to significantly increase antioxidant activity (GPX and CAT) under drought stress suggests a limited ability to cope with oxidative damage. This lack of upregulation in antioxidant enzymes likely contributes to their higher susceptibility to oxidative stress, impairing their overall stress response (Neto et al., 2010).

H_2_O_2_ content and MDA estimation are used as indicators of membrane damage following stress situations and subsequent ROS and free radical production (Mayne, 2013). Due to uncontrolled increases in free radicals, membranes become leaky and lipid peroxidation occurs through lipoxygenase activity, further contributing to free radical generation. As a result, fatty acids are damaged, producing hydrocarbon fragments like MDA. In our experiments, H_2_O_2_ and MDA concentrations in roots increased during water stress in both resistant and sensitive plants, showing levels nearly double in the sensitive those in the resistant clone. The higher levels of oxidative damage (MDA and H_2_O_2_) in sensitive clones suggest membrane destabilization. Increased lipid peroxidation, as indicated by higher MDA levels, can compromise the integrity of cell membranes, leading to impaired cellular function and increased susceptibility to further stress. This loss of membrane stability contributes to the overall reduced resilience of sensitive clones under drought conditions (Sairam and Saxena, 1999). This has been previously observed in pistachio leaf and root tissues under drought stress (Khoyerdi et al., 2016; Pakzad et al., 2019). It was proposed that regulating the activity of these enzymes during early growth stages could enhance a plant’s resistance to environmental stresses (Moussa et al., 2008).

A FTIR-ATR analysis was also performed to study the different responses to dehydration stress. Complex biological matrices, such as plant tissues and their extracellular gel-like composites, cannot be easily characterized using infrared (IR) spectroscopy of organic functional groups. The IR outputs primarily correspond to the main biological macromolecules, providing few vibrational signals. The technique is useful to read simplified common regions: polysaccharide moieties (950-1450 cm^-1^), peptide, amide and carbonyl/carboxyl planes (e.g., ester-bearing pectins or functional groups from cutin moieties, 1550-1800 cm^-1^), aliphatic chains with symmetric and asymmetric stretching signals (2750-2990 cm^-1^), accessory amines (3100-3300 cm^-1^) and hydroxyl intense signals (e.g., water, phenolic, polyphenolic and catechol, 3450 cm^-1^) (Schulz and Baranska, 2007). Unfortunately, this technique, which generally offers information about the functional groups of specific compounds without any selectivity related to the matrix, did not provide further insights into the biochemical or macromolecular differences between the resistant and sensitive genotypes. These data are usually analyzed using molecular biology techniques.

Drought stress tolerance is a complex trait that requires the involvement of several genes to ensure the survival of the plant in adverse conditions. Among other things, drought stress causes the plant cells to dry out and impairs the structure of the cell membranes. Due to its high hydration ability, dehydrin, a member of the LEA-II family, can prevent excessive water loss and maintain the membranes hydration protection system (Allagulova et al., 2003). The results obtained in studies of the DHN gene family in several species, broadly support the role of DHNs in stress tolerance (Velasco-Conde et al., 2012; Falavigna et al., 2015; Hassan et al., 2015; Hussain et al., 2015; Abedini et al., 2017; Li et al., 2023). These results are consistent with our results showing an over-expression of the DHN gene in the pistachio roots of drought-stress plants compared with control. Moreover, the increased levels of DHN protein or DHN transcript in drought-resistant hybrids, revealed by proteomic and transcriptomic studies, support the data obtained comparing the drought-tolerant clonal hybrid C9-4 and the drought-sensitive clonal hybrid C4-2 (Liu et al., 2015; Abedini et al., 2017; Ghatak et al., 2017). This significant upregulation in the resistant clone suggests that DHN plays a crucial role in enhancing cellular protection mechanisms against the deleterious effects of drought, contributing to the drought resilience observed in the resistant clone.

The *CDPK* gene plays an important role in the transmission of cellular Ca^2+^ signaling. Most *CDPK* genes are commonly expressed in organisms. However, some *CDPK* genes are expressed only in specific tissues or are induced by hormonal, biological, or abiotic stress conditions such as salt and drought stress (Sheen, 1996; Patharkar and Cushman, 2000). Furthermore, overexpression of these genes has been shown to increase drought stress resistance in various species (Saijo et al., 2001; Mittal et al., 2017; Mahmood et al., 2019). The increased expression of *CDPK* observed in our study suggests that CDPK is actively involved in calcium-mediated signal transduction pathways that may enhance stress tolerance mechanisms, particularly in the resistant clone. The upregulation of *CDPK* in the resistant rootstock suggests a more efficient or robust signaling cascade in response to drought stress. Similarly, the increased transcript level of *ZEP* genes detected in this work confirms their involvement in drought stress responses and the resistance mechanism in agreement with the literature (da Silva et al., 2022; Changan et al., 2023).

The expression of *DHN* can be influenced by CDPK activity. CDPKs, as part of the calcium signaling pathway, can phosphorylate transcription factors or other proteins that regulate the expression of stress-responsive genes, including *DHN*. This means that the activation of CDPKs in response to increased calcium levels during drought stress can lead to upregulation of *DHN* expression. This cascade enhances the plant’s ability to protect cellular structures and maintain function under stress conditions (Patharkar and Cushman, 2000; Liu et al., 2015). Minor differences in *ZEP* gene expression under both normal and drought conditions in resistant and sensitive pistachio cultivars suggest that the regulation of ABA biosynthesis at the ZEP level is not the main differentiating factor in the response to drought in these plants. However, the entire ABA metabolic pathway may continue to play a significant role in drought response.

With this work, we can confirm the role of these genes in pistachio species, which have so far only been studied in relation to salt stress (Moazzzam Jazi et al., 2017). In the future, a more comprehensive study involving more genotypes, tissues and genes could shed more light on the complex mechanism of drought-stress resistance in pistachio and allow the identification of new genotypes that are better adapted to adverse weather conditions.

## 5 Conclusion

The results of the study indicate that resistant clonal hybrids of Pistachio have well-integrated defense mechanisms that help to reduce damage caused by drought stress. This research provides insights into the regulatory processes of drought tolerance in pistachio plants, specifically in relation to root structure, osmolytes content, antioxidant activity and gene expression. These findings also provide clues for the breeding of drought-resistant cultivars and the selection of rootstocks suitable for drought-prone areas. Given the increasing use of clonal rootstocks for their advantages in terms of disease resistance, stress tolerance and overall performance, the C9-4 clonal rootstock could be a suitable option for areas with limited water availability.

## Data Availability

The datasets presented in this study can be found in online repositories. The names of the repository/repositories and accession number(s) can be found in the article/**Supplementary Material**.
